# Advancements in Functionally Graded Polyether Ether Ketone Components: Design, Manufacturing, and Characterisation Using a Modified 3D Printer

**DOI:** 10.3390/polym15142992

**Published:** 2023-07-10

**Authors:** Eric McNiffe, Tobias Ritter, Tom Higgins, Omid Sam-Daliri, Tomas Flanagan, Michael Walls, Pouyan Ghabezi, William Finnegan, Sinéad Mitchell, Noel M. Harrison

**Affiliations:** 1 College of Science and Engineering, University of Galway, H91 TK33 Galway, Irelandtom.higgins@nuigalway.ie (T.H.); omid.samdaliri@universityofgalway.ie (O.S.-D.); william.finnegan@universityofgalway.ie (W.F.);; 2 Ryan Institute for Environmental, Marine and Energy Research, University of Galway, H91 TK33 Galway, Ireland; 3 Éire Composites Teo, Údarás Industrial Estate, An Choill Rua, Inverin, Co., H91 Y923 Galway, Ireland; t.flanagan@eirecomposites.com; 4 CTL Tástáil Teo, Údarás Industrial Estate, An Choill Rua, Inverin, Co., H91 Y923 Galway, Ireland; m.walls@ctlcomposites.com; 5 Construct Innovate & SFI MaREI Research Centre, University of Galway, H91 TK33 Galway, Ireland; 6 I-Form, the SFI Research Centre for Advanced Manufacturing, D04 V1W8 Dublin, Ireland

**Keywords:** 3D printing, functional graded materials, PEEK, additive manufacturing, fused deposition modelling, material extrusion

## Abstract

Functionally Graded Materials represent the next generation of engineering design for metal and plastic components. In this research, a specifically modified and optimised 3D printer was used to manufacture functionally graded polyether ether ketone components. This paper details the design and manufacturing methodologies used in the development of a polyether ether ketone printer capable of producing functionally graded materials through the manipulation of microstructure. The interaction of individually deposited beads of material during the printing process was investigated using scanning electron microscopy, to observe and quantify the porosity levels and interlayer bonding strength, which affects the quality of the final parts. Specimens were produced under varying process conditions and tested to characterise the influence of the process conditions on the resulting material properties. The specimens printed at high enclosure temperatures exhibited greater strength than parts printed without the active addition of heat, due to improved bond formation between individual layers of the print and a large degree of crystallinity through maintenance at these elevated temperatures.

## 1. Introduction

The combination of traditional manufacturing methods for polymers and composites with additive manufacturing has revolutionised the production landscape. Traditional manufacturing processes such as hand lay-up, vacuum assisted resin transfer moulding, injection moulding, autoclave, extrusion, and compression moulding have long been employed for large-scale fabrication of polymers and composites parts [1,2,3]. These methods offer high production rates, precise control over material properties, and cost-effective solutions. However additive manufacturing, also known as 3D printing, has emerged as a production technique that complements traditional manufacturing processes. Additive manufacturing enables the rapid creation of complex geometries and customised designs with minimal material waste, making it ideal for prototyping as well as scaled-up production [4,5,6,7,8]. By combining traditional manufacturing methods with additive manufacturing, manufacturers can harness the advantages of both techniques, achieving greater flexibility, improved efficiency, and enhanced product innovation in the realm of polymers and composites. Additive manufacturing also enables the fabrication of space-grade components, necessitating desirable thermal and mechanical properties to withstand extreme environmental conditions [9,10]. In topology optimisation, complex geometries can be produced which are difficult to manufacture using standard processes but often can be fabricated using additive manufacturing technology [11]. There are many studies regarding the optimisation of additive manufacturing parameters in polymer composites to fabricate quality parts with appropriate mechanical properties and performances [12,13,14,15,16,17].

Instead of homogeneous or uniform material properties (e.g., stiffness, yield strength), Functionally Graded Materials (FGMs) have heterogeneous material properties within a single part vis-à-vis an intended locally varying microstructure, composition, or porosity [18]. Thus an FGM part is described as a component in which the properties change with respect to position [19]. Currently, a standard homogeneous part can be converted post-process into a limited FGM by using a dynamic heat-treatment procedure. Other methods introduce the graded microstructure by varying the alloy or polymer constituents across a part pre-process before solidification e.g., hot-press sintering bulk powder beds consisting of layers of different powdered alloys or polymers. Semi-crystalline polymeric materials are suitable for FGM as they offer the potential to locally vary the crystallinity (through temperature) control thus inducing varying properties across the part. With FGM design and manufacture a single solid part could exhibit different properties throughout its geometry. For example, a part may be case hardened on the outside for wear resistance (at the expense of being brittle) while retaining a more ductile interior. This would be an example of a thermal process being used to induce an FGM property in a part of a single homogenous material [20]. In polymer materials, the higher the degree of crystallinity (%), the higher the elastic modulus, strength and resistance to aggressive chemicals. A faster cooling rate means a low crystallinity and thus a more amorphous polymer is produced. A slower cooling rate and high pressure increase the chances of spherulite formation [21].

Multiple categories of additive manufacturing offer the potential for FGM manufacturing. Material extrusion (MEX) is especially suitable due to the ability to tightly control and modify the layer-by-layer process parameters conditions to manipulate the microstructure of the printed part as it gradually grows in the build direction [8,22,23]. Semi-crystalline polymers experience some distinct features that set them apart from their amorphous counterparts and can present difficulties regarding their processing [24]. Key MEX process parameters i.e., bed temperature, nozzle temperature, chamber temperature, print speed, nozzle diameter, layer thickness, and filling density determine the strength of the produced part [25]. The temperature parameters can be regarded as the most imperative factors to printing success due to their role in the crystallisation process. All other factors can be tuned with repeated printing and refinement to optimise the print and minimise production artefacts. During the MEX process, the filament is rapidly heated above its melting temperature, deposited, and then allowed to cool. During cooling, crystal formation occurs between the glass transition temperature and the melt temperature of the polymer. The high melting temperature of polyether ether ketone (PEEK) materials is the main difficulty in MEX printing, causing parts to undergo a large temperature change during printing, and thus can lead to significant thermal stress-induced deformation [26]. Geng et al. [27] have assessed the effects of printing speed and the extrusion speed on the dimensions of an extruded PEEK filament in 3D printing as well as the microstructure. The crystallinity of PEEK could be controlled through three main MEX process measures; ambient temperature control; nozzle temperature; and post-processing treatments [28,29,30]. The functional gradient was achieved with varying degrees of success with the aforementioned heat treatment methods. Furnace cooling and annealing were found to be the most effective methods resulting in the highest crystallinity of the part. The ambient temperature was maintained at approximately 200 °C. While the functional gradient was achieved, the additional post-processing step is time and energy intensive. Saini et al. [31] have studied the critical MEX processing parameters of PEEK 3D printing such as nozzle, chamber, and bedplate temperature and analysed their influence on the spinal fusion cages’ mechanical and thermal properties. Qin et al. [32] investigated the influence of the surface energy, polarity, and morphology of the treated carbon fibres on the impregnation behaviour and interfacial properties of CF/PEEK composites.

As reported in the literature, successfully manufacturing (non-FGM) PEEK parts via MEX is not a menial task, with FGM fabrication adding further challenges. Limited literature exists concerning optimised MEX parameters for PEEK, hence, an iterative approach to parameter control had to be used to refine the process. This work seeks to develop a low-cost 3D printer to manufacture FGM PEEK parts without post-processing by using localised heating methods in parallel to the MEX process that will act on the filament as the part is printed. Currently, FGM parts can technically be made with a 3D printer by varying the geometry and volume of the infill, and through printing with multiple materials. The novelty of this work is to introduce a functional gradient in the part by varying the material properties of a single homogenous material. Scanning electron microscopy (SEM) was conducted to assess the interaction of individually deposited beads of material during the printing process and the porosity level in the printed samples. To characterise the influence of the process conditions on the resulting material properties, tensile tests and hardness tests were implemented.

## 2. Materials and Methods

### 2.1. Aim and Objectives

The overall aim of this study was to create an affordable 3D printer that can produce FGM PEEK components using localised heating techniques, eliminating the need for additional processing steps.

However, to achieve this aim, the following steps were completed:

- SEM analysis was performed to evaluate the correlation between individual material beads during printing and the level of porosity in the printed samples.
- Mechanical tests (tensile and hardness tests) were performed to understand how the process condition affects the properties of the material.
- A crystallinity assessment of test samples 3D printed with different configurations was investigated.
- A demonstrator was 3D printed.

### 2.2. Materials and 3D Printing

FGM material extrusion requires a semi-crystalline polymer to vary the material properties. In this work, PEEK polymer was chosen as it exhibits high crystallinity across a relatively wide temperature range thus offering scope for altering material properties. PEEK also exhibits high strength and biocompatibility which is favourable given that one of the work’s main applications will be targeted towards the orthopaedic sector. As a new approach, this work seeks to manufacture FGM PEEK parts without post-processing by using localised heating methods that will act on the filament as the part is printed.

In general, self-assembly 3D printers do not offer all thermal processing conditions to be controlled and varied throughout the print. Therefore, in order to achieve this capability, the introduction of one or more of the following concepts is required (hot air delivery, dual nozzle design, ambient temperature control and enclosure, heated plate, and supplementary infrared heaters).

The summary of the above-mentioned concepts is listed in Table 1 including their advantages and disadvantages of each concept.

A decision matrix was created to assign a score to the different concepts put forward to determine the best concept moving forward to the final design. The heated plate and infrared bulbs for localised heat application scored the highest. The final design utilised what was learned in the research and conceptualisation of these design options to create a hybrid option wherein infrared heaters were used in a similar fashion to the heated plate.

A Velleman K8200 (Velleman Group, Havere, Belgium) 3D printer kit was purchased and developed to extrude PEEK filament and, subsequently, to develop PEEK FGM capabilities. A Velleman K8200 printer allows for the extrusion of low-performance, low-melting point polymers such as ABS and PLA (less than 190 °C). To facilitate the extrusion and FGM of PEEK, the purchased kit required extensive modification to reach the required temperatures for printing PEEK filaments (approximately 400 °C). The required modifications were carried out in a stage-by-stage process as part of an iterative final solution. As part of the PEEK extrusion and FGM process, a localised heat source was required to thermally affect the printed parts, therefore infrared lights were added to the print chamber. The stock hot end heater cartridge on the K8200 can reach a maximum operating temperature of 190 °C, so the stock hot end was replaced with a purchased all-metal “V6 hot end” (E3D, Oxfordshire, UK). Owing to the expected heat flow through the new aluminium hot end through to the extruder assembly, cooling features were added to the hot end. In order to avoid steep temperature differentials and associated detrimental effects, a printing bed with an operating temperature closer to the extruded PEEK temperature (approximately 400 °C) was required. Concurrently, the temperature of the bed must facilitate printing at higher temperatures to assist with crystallinity growth at a temperature capability above PEEK’s glass transition temperature (Tg = 143 °C).

**Figure 1:**
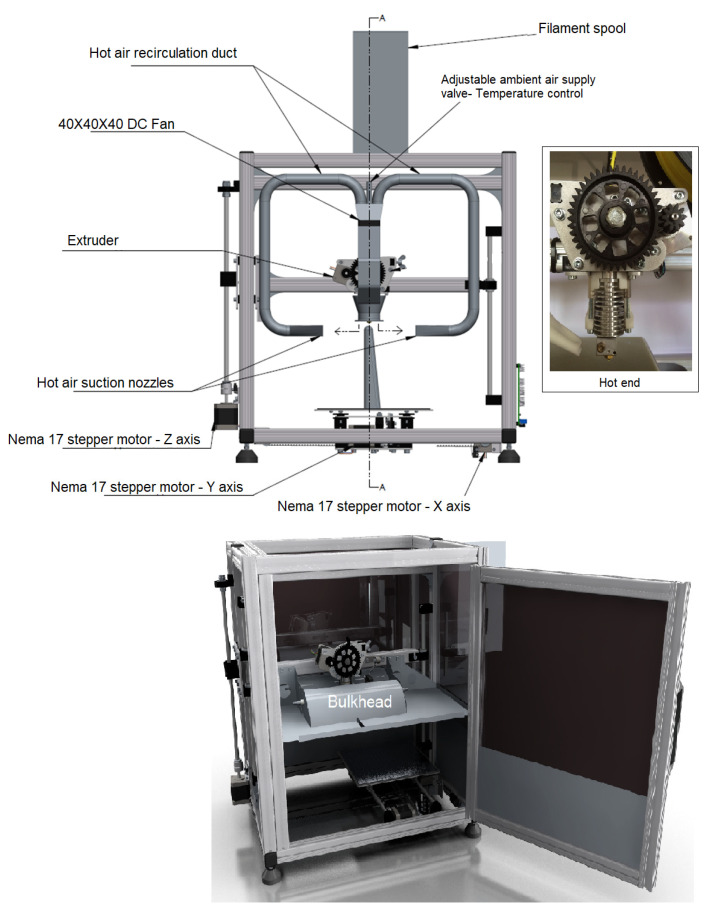
The modified and developed 3D printer.

The stock K8200 hot bed was capable of a maximum operating temperature of 60 °C, therefore a high temperature 500 W bed with a maximum operating temperature of 200 °C was procured and fitted to the existing x-axis carriage. To reduce the energy required to control the process temperatures, a reduced volume was accomplished by means of creating an enclosure for the rig. Due to PEEK being a hygroscopic material, it has to be stored in a dry environment. For this reason, an electrical junction box with an IP65 rating was purchased and converted into a filament storage box. To reduce the likelihood of motor failure, an Arduino-based-temperature-sensor data logger was built, wherein TMP37 temperature sensors were soldered to a shielded wire and attached to the sides of the motors (Figure 1).

The main process parameters employed in the optimisation of the PEEK printing process to regulate the ambient temperature were infrared temperatures of 20–140 °C (+5 °C/−5 °C) and heated bed temperatures of 40–150 °C (+5 °C/−5 °C). Print speeds of 15 mm/s up to 60 mm/s and printing infill patterns were used to control the localised temperature. The glass plate on the heated bed had to be prepped prior to every print to ensure successful adhesion of the print part. A layer of the commercially available adhesive Pritt stick was applied to the glass surface. This improved bed adhesion since it provided a slightly rougher surface for the filament to adhere to when combined with a wide brim. The ideal offset brim thickness was found to be 12 mm for the dog bone specimen which was based on sufficient levels of bed adhesion and minimal wastage. The most important process variables are listed below in Table 2 which were empirically found to be the ideal printing conditions to influence high crystallinity formation while still producing acceptable working conditions and achieving minimal distortion and sufficient bed adhesion.

The base Repetier software for controlling the print parameters was insufficient for the tight process control that was required and hence the slicing software Cura 4.3 was utilised wherein parameters such as the brim width and the individual print speeds of the print perimeter and infill could be specified.

### 2.3. Testing Methodologies

The tensile test and Rockwell hardness test were carried out as a quantitative verification of material properties for different samples printed under different process conditions such as the ambient temperature in the enclosure. Qualitative assessment of samples was also carried out in the form of a scanning electron microscope (SEM) test. The SEM test shows the interlayer boundaries of a cross-section of a printed part as well as highlighting any porous regions or delamination that may have occurred due to the extrusion process. For each test, at least five specimens were printed for each of the following process conditions; cool (60 °C, i.e., no active applied heat), applied heat ambient of 120 °C, applied heat ambient of 140 °C.

#### 2.3.1. Tensile Testing

**Figure 2:**
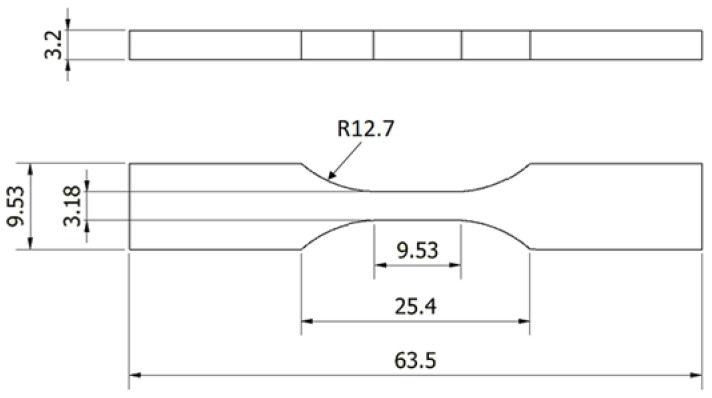
Tensile test specimen as specified in ASTM D638 (dimensions in mm).

Two types of tensile tests were carried out using an Instron 4467 machine. One traditional tensile test uses a dog bone specimen and a single strand (of filament) tensile test. The latter was carried out to determine the tensile properties of the filament before printing for comparison with the samples printed under different printing parameters and environmental conditions. The standard used for the dog bone tensile test was ASTM D638 as this is the typical standard used for testing the tensile strength of polymers (Figure 2). The standard used for the single-strand tensile test was ASTM D2566.

The tensile tests were carried out in environmental conditions of 50% humidity and 23 °C. Different PEEK samples were tested under different conditions for the baseline prints, i.e., no additional heat treatments from the printer.

The tensile-tested specimens were investigated to determine the loss of mechanical performance due to deformities of porosity and interlayer adhesion as the main influencers of structural integrity in material extrusion printed parts. Equation (1) below shows the direct correlation of the porosity levels to the resulting ultimate tensile strength. Due to the effects of porosity on the reduction of sample density, Equation (2) was used to calculate the subsequently reduced Young’s Modulus, E [33]. P is porosity, UTS_0_ is the ultimate tensile strength with zero porosity, and n is a power law constant.

(1) $$UTS={UTS}_{0}\;e^{-nP}$$

(2) $$E=E_{0}(1-1.9P+0.9P^{2})$$

#### 2.3.2. Hardness Testing

A hardness test was carried out using an Avery Denison Rockwell hardness apparatus with a diamond indented to determine the hardness of multiple different samples printed to determine whether a change in hardness was achieved through different processing conditions, i.e., percentage crystallinity. The specimen, as per the standard (ASTM D875), was required to be at least 20 × 20 × 6 mm^3^. Three different types of printed samples were tested, one with a low ambient temperature (45 °C) and two samples with applied heat with an ambient temperature of 120 and 140 °C, respectively.

#### 2.3.3. SEM Testing

**Figure 3:**
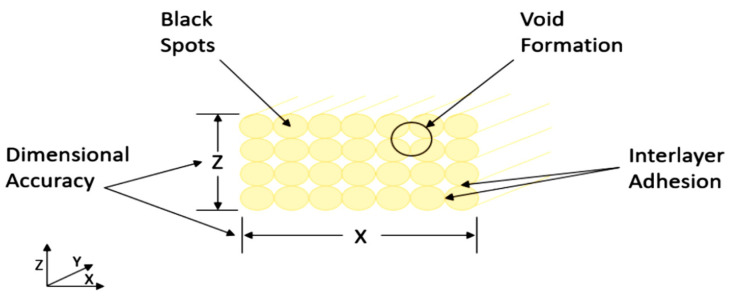
Scanning Electron Microscopy microstructure formations.

Scanning Electron Microscopy (SEM) was carried out using Hitachi S-4700 Scanning Electron Microscope (Hitachi, Tokyo, Japan) on extruded PEEK parts with the purpose of characterising the microstructural formations. SEM Tests were carried out to examine the correlation between the thermal processing parameters in the extrusion rig to the materialisation of four key microstructural formations as listed in Figure 3.

**Figure 4:**
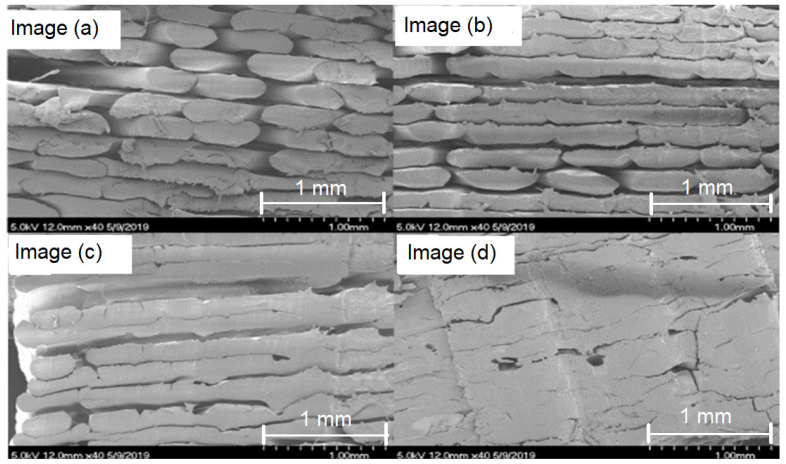
Scanning Electron Microscope images taken at × 40 magnification levels, (**a**) Nozzle temperature = 380 °C, Chamber temperature = 55 °C, Bed temperature = 125 °C, Print speed = 40 mm/s, Air Flow = 80%, (**b**) Nozzle temperature = 380 °C, Chamber temperature = 55 °C, Bed temperature = 125 °C, Print speed = 40 mm/s, Air Flow = 70%, (**c**) Nozzle temperature = 390 °C, Chamber temperature = 120 °C, Bed temperature = 125 °C, Print speed = 40 mm/s, Air Flow = 80%, (**d**) Nozzle temperature= 390 °C, Chamber temperature = 120 °C, Bed temperature = 135 °C, Print speed = 40 mm/s, Air Flow = 80%.

Figure 4 shows the results of SEM on a range of samples extruded at various process parameters. It can be concluded that the effects of the ambient temperature around the printed parts and printing speed play a key role in the formation of voids at a microstructure level.

To quantify the levels of porosity in the SEM images, the “ImageJ (Fiji)” processing program was used. It can be seen from the above images (a)–(d), that the porosity levels have been reduced from a level of 39% in sample image (a) to a level of 4% in sample image (d).

### 2.4. Crystallinity Assessment

Two different prints were conducted and tested, a low crystallinity part and a high crystallinity part (Table 3).

**Figure 5:**
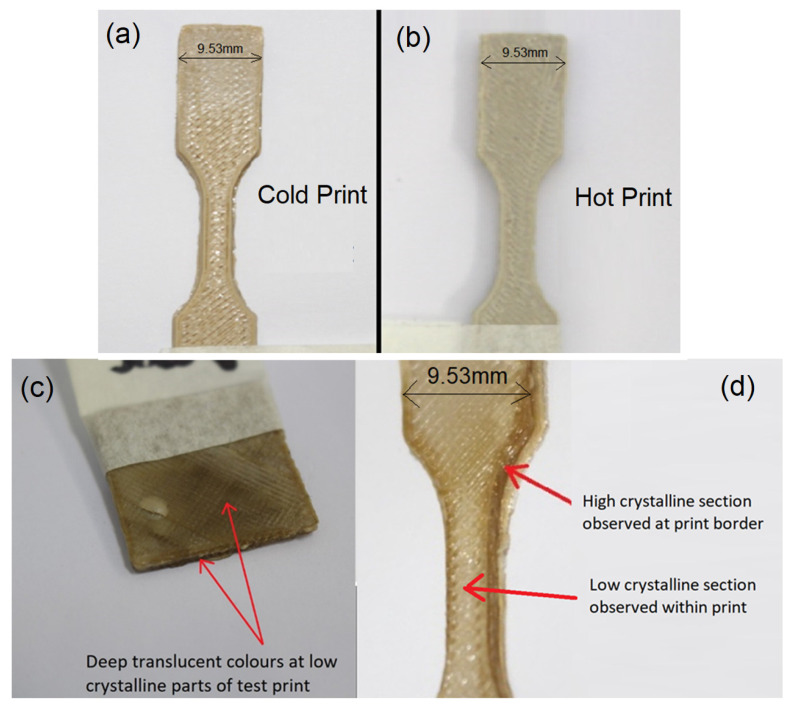
Colour variation between the “Cold” print and the “Hot” print.

Upon reviewing the test specimens printed under the process parameters described in Table 3, they both showed good colouration indicative of crystallinity growth. The slight variation in colour is shown in Figure 5a,b.

However, when compared to truly amorphous prints, the colour for a low crystalline print should be much deeper and translucent. This contrast is displayed in Figure 5c,d where, on the left-hand side of the figure, is a part printed with a fan directed towards the part in order to increase cooling and on the right-hand side (RHS) is another “cold” print test specimen.

**Figure 6:**
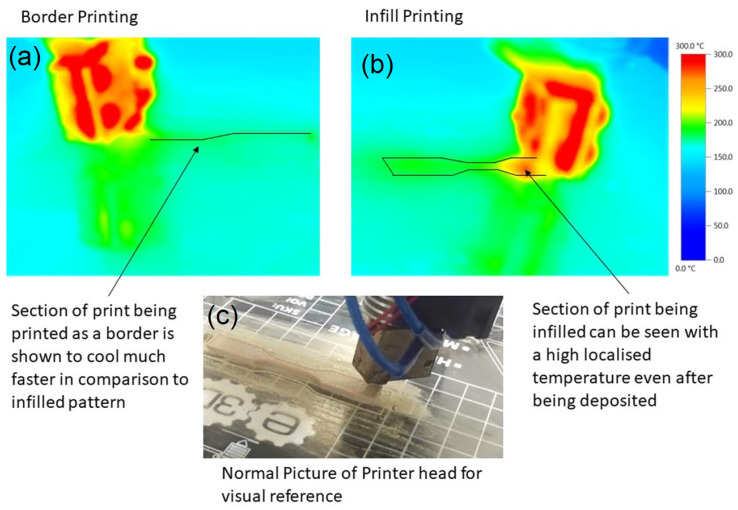
Thermal imaging camera pictures of different printing conditions.

Comparing the images in Figure 5, it is clear that the “cold” test specimens were not fully amorphous. To explain the difference in crystallinity observed in Figure 5d, it was deduced that the infill pattern of the printer introduced enough heat into the centre of the part to effectively crystallise it, but did not do so when laying down the border at the start of each layer. The presence of the hot end in the vicinity of the beads when they are placed alongside each other for a relatively close amount of time may also have a large effect on the cooling rate. Referencing back to Figure 5c,d, this describes how that print part developed a border of a low crystalline region. To investigate the effect of the infill pattern and the proximity of the hot end to the part, a thermal imaging camera was used to observe the printing process. Figure 6 shows the temperature that the filament cools to as soon as it is laid. As hypothesised, the border effectively cools much quicker than the infilled pattern. The residual heat left behind by the proximity of the hot end relative to the previously laid beads and the laying of hot material alongside the beads is enough to fully crystallise an area and potentially recrystallise an area below it.

## 3. Results and Discussion

The design must possess the capability to print parts of varying crystallinity while not adversely affecting mechanical properties such as porosity and interlayer bonding. Important printing improvements were identified during the testing and optimisation process detailed as follows. A self-levelling bed was deemed to be a necessary upgrade as it would improve the first layer adhesion and accuracy. It would also reduce the setup times at the start of the process as the bed can lose its positioning through x−y axis movements, thus requiring releveling which is a time-consuming process. This is already a standard item on most printers capable of printing PEEK. Since the current design allows for the movement of the x and y axes, it is not practically possible to enclose the base of the heated chamber. A method of relocating the movement of the x- or y-axis to be combined with the z-axis would allow the base of the machine to be sealed, thus allowing for a more controlled ambient temperature to form higher-quality parts. An independent system for actively cooling the print was identified as a requirement to accelerate cooling rate when needed. The application of high temperatures was effectively accomplished using infrared heating elements. To concentrate the heat energy onto parts, a parabolic reflective surface would serve to focus the IR light onto the print part. Future control of the localised temperature will facilitate tighter FGM manufacturing tolerances and would create improved repeatability. The single-strand test was carried out to characterise the supplied PEEK filament, and through the test, it was found that the strands had a UTS of 91 MPa, which is in line with theoretical values for PEEK. The experimental results from tensile tests are summarised in Table 4.

The initial batch of PEEK dog bone specimen having exhibited minimal warping, suffered from poor inter-laminar bonding which resulted in the premature failure of the specimen and a large degree of scatter between the results. This could also have been caused by porous regions within the print, signalling a need for SEM analysis to gain a deeper understanding of the effect of processing conditions on the material structure and hence the mechanical properties.

The second set of samples tested was processed under an elevated and sustained ambient temperature of 120 °C. The results for the UTS of these samples are in the range of 43.4–50 MPa, which little variation, demonstrating repeatability in the print quality. The discrepancy in these results, when compared to the UTS of a solid piece of PEEK, can likely be attributed to minor porous regions due to the print process which are sites for cracks to grow and propagate resulting in premature failure. Reduced strength could also stem from the poor fusion of the respective layers in the component. The last set of tensile specimen tested were manufactured under an ambient enclosure temperature of approximately 140 °C. There are two outliers in this set of data represented by samples H7 and H9, respectively, while H10 demonstrated similar strengths to the samples manufactured at 120 °C. The two outliers can likely be explained by manufacturing defects, in the form of slight warping which caused cracking while loading the specimen in the jaw clamps of the tensile test rig, reducing the final failure load. Table 4 presents the tensile strengths of the components printed under different ambient conditions. From the graph, it is clear that the parts printed at 120 °C resulted in the least scatter, whereas the samples printed at 55 °C were the most varied, mostly due to inconsistent part quality due to different printing parameters. The maximum tensile strength of the samples tested was found to be approximately 50 MPa, significantly less than the tensile strength of PEEK of approximately 90 MPa. This discrepancy in strength is likely due to pores or reduced layer adhesion resulting in a significantly less solid part than one machined from a sheet of PEEK. This reduced strength could also be attributed to the +45/−45° infill pattern used.

Initial samples experienced large degrees of warping, this issue was fixed for subsequent builds where further steps were taken to reduce the air flow from the cooling fan into the build chamber. This caused warping due to the large thermal gradient experienced by the part resulting in residual stresses. The warped parts were unsuitable for tensile testing as fixing them in the tensile tester jaw clamps damaged the parts prematurely. The second set of parts printed with applied heat at 120 °C did not experience this warping and hence could be tensile tested effectively.

Three samples were produced for the Rockwell hardness test, printed at ambient temperatures of 45, 120, and 140 °C. The results of hardness testing are presented in Table 5.

The specimen printed at 45 °C displayed the lowest hardness on the Rockwell scale at 49.2, with the sample printed at 120 °C exhibiting the highest strength of the samples tested. The samples were tested against a cut-off sample from a roll of PEEK which was found to have a Rockwell hardness within the theoretical range of 90–100, with an average of 92.9. Hardness in polymeric materials represents a high degree of crystallinity. This is confirmed by the sample printed at ambient which visually exhibited low crystalline properties due to the translucent areas, appearing darker on the surface of the sample as seen in Figure 5.

From the results of the SEM test, seen in Figure 4, the structure of the specimen tested varies significantly (Table 6). In the order of (a)–(c), the samples were printed in increasingly higher temperatures, finally resulting in a porosity level of 4%, as seen in Figure 4d. Using the calculated porosities, the expected tensile strengths of the samples were calculated, however, there was still a large degree of error between the tested results and the predicted results. This suggests that porosity was not the only factor to have detrimental effects on the strength. Furthermore, not seen in these pictures is the level of bonding of the outer perimeter to the infill, wherein poor bonding could result in reduced strength to failure as with the aforementioned delamination failure.

**Figure 7:**
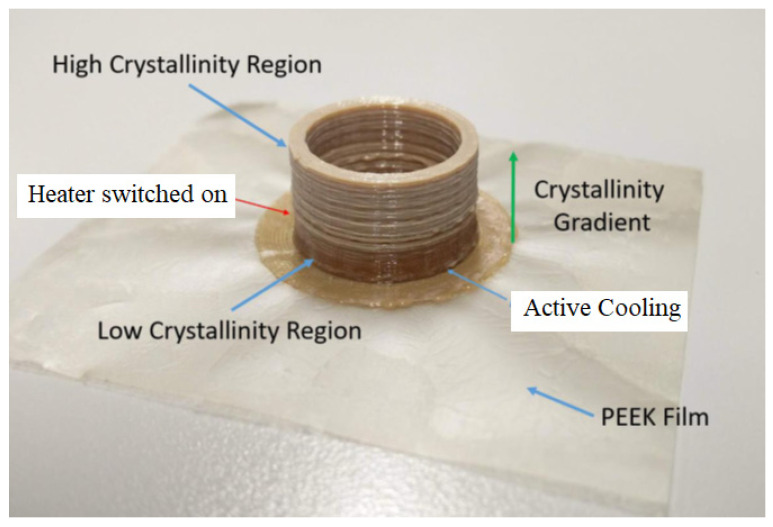
Crystalline gradient printed on the modified printer shows clearly the low crystalline and high crystalline regions (Internal diameter = 26 mm, External diameter = 30 mm, and Height = 25 mm).

Figure 7 shows a functionally graded part printed on the final machine, the low crystalline/amorphous region can be clearly seen by the darker translucent material. The high crystalline section can be recognised by the lighter beige colour on the part. This part demonstrates the capability of the machine to print FGMs, realising one of the main objectives of this work. The low crystalline region was printed first and was actively cooled with fans as it was deposited. As can be seen from Figure 7, there is a transition from the low crystalline region to the high crystalline region which is representative of when the heater was switched on. This part is representative of the capabilities of the modified and optimised machine and the lessons learned from producing this part can be applied to components with high functionality in the future.

## 4. Conclusions

This research has focused on the development of an affordable 3D printing method for manufacturing FGM PEEK parts without the need for post-processing. By employing localised heating methods that act directly on the extruded filament during the printing process, it becomes possible to achieve functional gradient properties within a single part. This approach represents a significant advancement compared to existing techniques that rely on varying infill geometry or using multiple materials. As in this research, a modified and developed 3D printer was presented. Future steps of this advanced MEX development would be to add a capability of 3D printing of short fibre and continuous fibre composites to the modified 3D printer. The primary conclusions of this study are:

- Several optimisations have been conducted to able a low-price/low temperature 3D printer to print high temperature polymers such as PEEK materials.
- FGM capability of the modified machine was proven and achieved through the control of temperature within the build environment as demonstrated. The functional gradient in a single part was achieved in a novel manner by the application of heat treatment during the manufacturing process removing the requirement for post processing.
- Parts printed at high enclosure temperatures exhibited greater strength than parts printed without the active addition of heat via the heater, due to improved bond formation between individual layers of the print and a large degree of crystallinity through maintenance at these elevated temperatures.
- It was measured that the maximum tensile strength of the PEEK specimens tested was approximately 44% less than the tensile strength of PEEK of approximately 90 MPa. This discrepancy in strength is likely due to pores or reduced layer adhesion resulting in a significantly less solid part than one machined from bulk PEEK.
- The specimen printed at 45 °C displayed the lowest hardness on the Rockwell scale at 49.2, with the sample printed at 120 °C exhibiting the highest strength of the samples tested.
- From the SEM results, it was found that the porosity of printed samples was at the level of 4%. Using the calculated porosities, the expected tensile strengths of the samples were calculated, however, there was still a large degree of error between the tested results and the predicted results.
- Effectively, the infill pattern and slicing of the physical specimen had a large effect on the resulting crystallinity of the produced parts.

## Figures and Tables

**Table 1:** Comparison of different concepts to control the temperature during printing.

Concept	Advantages	Disadvantages
Hot air delivery	Allows precise temperature control of extruded material. High inter-laminar shear strength is possible. Localised high temperature has a low risk of thermal damage to electronics. Can also actively cool the part.	May cause deformation on site due to forced air. Formation of bubbles on the surface is possible and potentially leading to unwanted pores. Air velocity may reduce the dimensional accuracy of print.
Dual nozzle design	No wait time when a higher extruding temperature is required (i.e., one extruder will always be at the correct temperature for the next phase of printing while the other extruder is in use). Compact design does not limit the build area. Off-the-shelf component can be used with modifications. Cost effective.	The heat from the “hot” head may be too high and influence the “cold” printing. Unsure of the underlying assumption of there being enough thermal energy in the extruded “hot” material.
Ambient temperature control and enclosure	Cost effective. Exact control over ambient conditions. Active cooling can be achieved.	May not provide fine enough control. Isolation of electronics from high ambient temperatures may prove difficult. Can only achieve functional gradient in one axis.
Supplementary Heated Plate	Inter-laminar shear strength increased from localised heat at the print site. Easy to control as very few components are required. No moving parts. Localised high temperature has a low risk of thermal damage to electronics.	Cannot affect previous layers, only the top layer. Limited by convection. Can only achieve functional gradient in one axis.
Infrared Lamp	Precise control of crystallisation in two axes. Easy control of temperature using a microcontroller. Even distribution of heat is possible. Easy to test and implement (i.e., off-the-shelf components).	No active cooling. Can only achieve functional gradient in one axis.

**Table 2:** Print Parameters used to print test specimen.

Bed Preparation	Cleaned after each use and a thin layer of Pritt stick applied
Adhesion Type	Brim (12 mm)
Print Speed	20 mm/s
Outer Perimeter speed	10 mm/s
Inner Perimeter Speed	10 mm/s
Infill Speed	20 mm/s
Infill Pattern	+/−45°
Infill	100%
Flow Rate	75%
Extruder Temperature	390 °C
Bed Temperature	120–140 °C

**Table 3:** Thermal processing conditions for two prints.

	Test 1	Test 2
Bed temperature (°C)	120	140
Ambient air temperature (°C)	50	140
Radiation temperature (°C)	N/A	150
Nozzle temperature (°C)	390	390

**Table 4:** UTS Results for different PEEK samples tested. (Average values for 3 replicates).

Sample	Extruder T °C	Bed T °C	Enclosure T (°C)	Bulkhead	Air Flow	Measured UTS (MPa)
1	370	120	55	No	65%	24.08
2	380	125	55	No	70%	29.85
3	380	125	55	No	80%	32.70
4	370	120	55	Yes	80%	31.97
5	380	120	55	Yes	80%	36.44
6	390	125	55	Yes	80%	46.65
H1	390	135	120	Yes	80%	49.87
H2	390	130	120	Yes	75%	43.39
H3	390	150	120	Yes	75%	47.57
H4	390	125	120	Yes	75%	43.67
H5	390	140	120	Yes	75%	47.44
H6	390	125	120	Yes	75%	47.10
H7	390	125	140	Yes	75%	30.07
H8	390	130	140	Yes	75%	47.07
H9	390	140	140	Yes	75%	32.09
H10	390	145	140	Yes	75%	48.40

**Table 5:** Hardness test results (Rockwell Hardness).

Sample	Condition	Average
1	Ambient: 45 °C	49.2 ± 1.2
2	Ambient: 120 °C, Bed: 150 °C	75.2 ± 2.3
3	Ambient: 140 °C, Bed 140 °C	57.4 ± 1.1
4	Rod Cut-off	92.9 ± 2.6

**Table 6:** SEM testing results of actual UTS and calculated UTS based on porosity levels.

Image	Sample Name	Adjusted Area	Tested UTS (MPa)	Calculated UTS (MPa)	Infrared Temperature (°C)	Bed Temperature (°C)	Print Speed (mm/s)
(a)	3	0.39539	32.7	13.84	55	125	40
(b)	2	0.17495	29.85	41.69	55	125	60
(c)	6	0.09052	46.65	63.59	55	125	40
(d)	H1	0.04234	49.87	80.92	120	135	40

## Data Availability

Publicly available testing data are available in this publication. Additional testing data may be granted through contact with the corresponding author.
